# Knowledge, Attitudes, and Practices Towards the Influenza Vaccine Among Pregnant Women: A Systematic Review of Cross-Sectional Studies

**DOI:** 10.3390/healthcare13111290

**Published:** 2025-05-29

**Authors:** Franciszek Ługowski, Julia Babińska, Jakub Kwiatkowski, Nicole Akpang, Aleksandra Urban, Joanna Kacperczyk-Bartnik, Paweł Bartnik, Agnieszka Dobrowolska-Redo, Ewa Romejko-Wolniewicz, Jacek Sieńko

**Affiliations:** 2nd Department of Obstetrics and Gynecology, Medical University of Warsaw, Karowa 2 Street, 00-315 Warsaw, Poland

**Keywords:** influenza vaccine, maternal vaccination, vaccine hesitancy, pregnancy, influenza, flu, vaccine uptake

## Abstract

**Background:** Influenza is an acute viral disease that primarily affects the airways. It is caused by influenza A and B—RNA viruses. The disease is associated with significant morbidity and mortality. The prevention of influenza includes chemoprophylaxis and vaccination, which are the primary preventive measures against influenza infection and should be highly considered by everyone during influenza season. **Methods:** A systematic literature search was performed in the databases of PubMed, Web of Science, Scopus, and Embase until September 2024. The review was conducted following the preferred reporting items for systematic reviews and meta-analyses (PRISMA) guidelines. **Results:** Eventually, a total of 20 publications were included in the final analysis of this systematic review. While general awareness of influenza was moderate, detailed understanding of complications and vaccine safety was frequently lacking. Misconceptions—such as fears of fetal harm and confusion between antiviral and antibiotic treatments—were widespread. Vaccine uptake was generally low but strongly correlated with receiving a healthcare provider recommendation. Willingness to vaccinate was higher in settings where participants were educated during the study process, indicating a crucial role of health communication. **Discussion:** According to the reviewed literature, the reluctance to receive maternal vaccination often stems primarily from fears or concerns about adverse reactions or misconceptions about the vaccine’s effectiveness, as well as the absence of a physician’s recommendation. Misconceptions regarding vaccine safety, limited understanding of influenza severity, and a lack of clear communication from healthcare professionals are key contributors to low vaccination uptake. Importantly, multiple studies confirmed that recommendation by a trusted healthcare provider significantly increases vaccine acceptance. **Conclusions:** These findings highlight the urgent need for targeted educational strategies, improved antenatal counseling, and systems-level support to ensure that maternal influenza vaccination becomes a standard and trusted component of prenatal care worldwide.

## 1. Introduction

Influenza is an acute viral disease, caused by influenza A and B viruses which are RNA viruses, which primarily affects the airways. Influenza B viruses are mostly found in humans; whereas influenza A viruses (IAV) cause annually recurring epidemic diseases and emerge from a zoonotic reservoir. Influenza A viruses undergo frequent antigenic drift, acquiring mutations that enable them to evade the host immune response [1,2,3]. The disease is associated with significant morbidity and mortality [1]. The Centers for Disease Control and Prevention (CDC) estimates that the seasonal influenza recurrence has led to approximately 9.3 to 41 million cases, 100,000 to 710,000 hospitalizations, and 4900 to 51,000 deaths annually in the United States [4]. Antiviral medications, such as neuraminidase inhibitors (oseltamivir, peramivir, or zanamivir) can reduce the risk of a severe disease if administered within 24 to 72 h after symptoms onset [5]. The prevention of influenza includes vaccination, which is the primary recommended preventive measure for the general population and particularly crucial during pregnancy. Antiviral chemoprophylaxis, while available, is reserved for specific high-risk individuals (pregnant women, adults aged 65 years and older, children under 5 years, individuals with chronic medical conditions, etc.) or institutional outbreak settings, in line with current public health guidelines [2]. The efficacy of the influenza vaccine varies significantly, ranging from 10 to 20% when the vaccine does not match the most common strain of the virus in the season up to around 60% if it effectively targets the circulating strain [6,7,8]. Pregnancy is associated with an increased risk of pneumonia and adverse perinatal and neonatal outcomes during influenza [9]. Influenza poses a particularly serious threat to pregnant women as they are more likely to suffer severe complications such as pneumonia, acute respiratory distress syndrome, and even death [9]. Physiological and immunological changes during pregnancy—including reduced lung function and altered immune response—contribute to this vulnerability. Furthermore, maternal influenza infection increases the risk of adverse perinatal outcomes, such as preterm birth and low birthweight [9]. Crucially, influenza vaccination during pregnancy not only protects the mother but also provides passive immunity to the infant. Vaccine-induced maternal antibodies cross the placenta and safeguard the newborn during the first six months of life, when they are too young to be vaccinated themselves [10]. This dual benefit underscores the essential role of maternal influenza immunization in protecting both maternal and infant health [11]. Most influenza vaccines are quadrivalent and stimulate the immune system against four antigens or four different strains of the influenza virus. Types of influenza vaccines include inactivated, live attenuated, and recombinant vaccines [2]. The American College of Nurse–Midwives and the American College of Obstetricians and Gynecologists both recommend that pregnant women receive an inactivated influenza vaccine (IIV) during flu season [12]. IIV is also recommended for all pregnant women, regardless of trimester, in the vast majority of European countries [13]. Moreover, breastfeeding women may safely receive an IIV as well. However, live vaccines are contraindicated during pregnancy and should be avoided [14]. Numerous studies have proven that influenza vaccines are the most effective strategy for preventing severe illness and complications of influenza infection [11,15]. Furthermore, vaccinated pregnant women transfer antibodies to their newborns through vertical transmission, providing essential protection of the newborn against influenza and reducing the risk of hospitalization during the first 6 months of life [10]. Moreover, maternal vaccination against influenza is not associated with any pregnancy complications, including small-for-gestational-age infants or preterm delivery [16]. Unfortunately, despite significant benefits and safety, the coverage of influenza vaccination among pregnant women is limited. Many women continue to believe, incorrectly, that the vaccine causes significant birth defects despite educational programs and awareness campaigns [17], and some healthcare professionals are reluctant to recommend or even suggest it [18]. Despite official recommendations and the demonstrated safety and effectiveness of maternal influenza vaccination, coverage rates remain suboptimal in many countries. In the United States, approximately 53.6% of pregnant women are vaccinated against influenza according to the CDC [19]. However, global estimates show wide variability. For example, uptake rates range from as low as 3% in some low- and middle-income countries to over 60% in high-income settings such as the United Kingdom and Spain [20,21,22]. This disparity highlights the influence of healthcare infrastructure, provider recommendation, socioeconomic factors, and vaccine accessibility on maternal vaccination rates worldwide. It is noteworthy that primary preventive measures are essential for preventing and managing influenza, particularly for pregnant women. This includes not only adherence to basic rules of hygiene but also vaccination during pregnancy. Hence, there is a need to raise the awareness of influenza during pregnancy. This study aimed to systematically assess and review pregnant women’s knowledge of influenza regarding infection, preventive measures, and complications, alongside their attitudes and concerns, and their intentions for vaccination. Previous systematic reviews on this topic were published in 2014 by Yuen et al. [23] and in 2021 by Adeyanju et al. [24]. However, our study presents the most up-to-date and comprehensive synthesis of cross-sectional studies published since 2015, encompassing diverse geographic and economic settings. Unlike previous reviews that primarily discussed general attitudes or focused on specific regions or qualitative findings, our review systematically quantifies knowledge levels, vaccine uptake, and factors associated with hesitancy using standardized inclusion criteria. We also include data from low-, middle-, and high-income countries, allowing for global comparisons and a more granular understanding of knowledge and practices related to influenza vaccination during pregnancy.

## 2. Materials and Methods

A systematic literature search was conducted across multiple databases, including PubMed, Web of Science, Scopus, and Embase, up to September 2024. This systematic review adhered to the guidelines outlined in the preferred reporting items for systematic reviews and meta-analyses (PRISMA) guidelines. Furthermore, the review was registered in the international prospective register of systematic reviews (PROSPERO) under the registration number CRD42024609635. The search strategy incorporated the following keywords: “knowledge” OR “attitude” OR “practice” OR “belief” OR “barrier” OR “perception” AND “pregnancy” OR “pregnant” OR “pregnant women” OR “maternal” AND “influenza” OR “flu” AND “vaccine” OR “vaccination” OR “immunization”. Additionally, reference lists of the included studies were manually reviewed to identify any further relevant studies.

The inclusion criteria specified that eligible studies had to be cross-sectional, published in English, and published since 2015. Studies were excluded if they employed different study designs, focused on diseases or vaccines other than influenza, were published in languages other than English, or were released before 2015. The risk of bias in the included studies was independently assessed by two researchers (F.Ł. and J.B.) utilizing the Downs and Black checklist 20. Only non-randomized studies that achieved a minimum score of 11 out of 13 were considered eligible. After the initial screening, the pre-selected studies underwent further evaluation to determine their final inclusion in the systematic review.

A standardized data extraction form was developed for this study. Data extraction was conducted independently by two researchers (F.Ł. and J.B.) and subsequently cross-verified. The extracted data included authorship, article type, year of publication, sample size, data collection method, and key findings related to influenza infection, primary preventive measures, potential complications, attitudes, and willingness to receive the vaccine. A narrative synthesis was performed to integrate methodological characteristics, such as study design, input data (including vaccine effectiveness and uptake), and assumptions regarding vaccine impact, alongside study findings and the overall quality of the included studies.

## 3. Results

**Figure 1:**
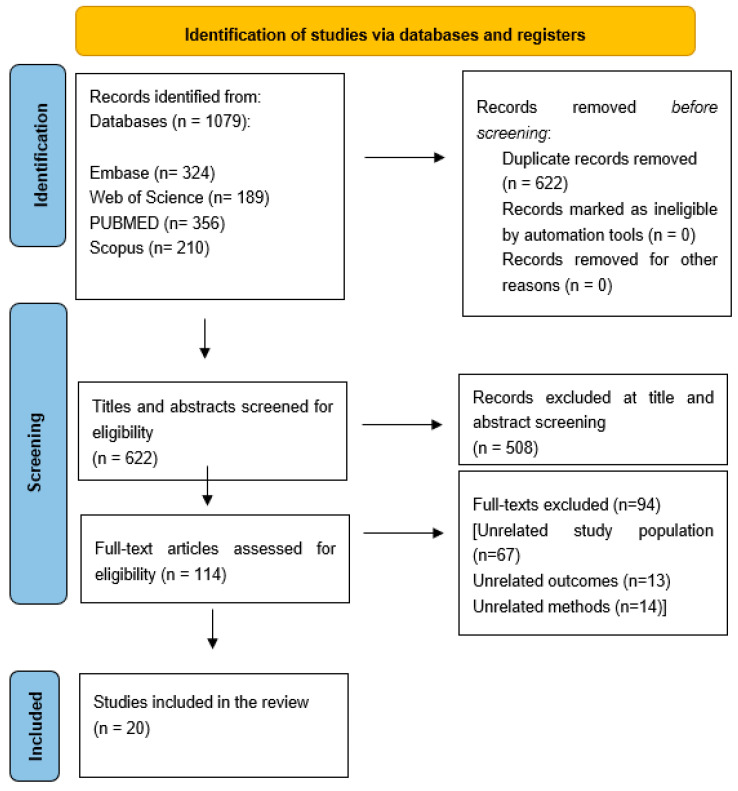
Prisma flow chart of the screening process.

A total of 1079 articles were identified through a systematic literature search (Figure 1). Following the initial screening, 457 duplicate records were removed and the remaining 622 titles and abstracts were assessed for eligibility. After the screening process, 114 full-text articles were selected for further evaluation, of which 94 were excluded based on the predefined eligibility criteria. Ultimately, 20 studies met the inclusion criteria and were incorporated into the final analysis of this systematic review (Table 1).

### Key Findings

Across the included studies, several important patterns emerged regarding pregnant women’s knowledge, attitudes, and practices toward influenza vaccination.

Knowledge Gaps: General awareness of influenza was moderate to high in many settings; however, detailed knowledge of transmission routes, complications during pregnancy, and vaccine safety was often poor. For example, while 90% of women in Singapore [29] knew that a virus causes influenza, 46% mistakenly believed antibiotics were the treatment of choice. Similarly, in Poland, 96.5% recognized influenza as a viral infection [37], yet only 51.1% were aware that antivirals are used for treatment.

Misconceptions about Vaccine Safety: A recurrent barrier to vaccination was concern about the vaccine’s impact on fetal health. In China, 86.3% of respondents believed influenza vaccination could harm the fetus [26], and in Saudi Arabia, only 13.1% believed it was safe during pregnancy [34].

Influence of Healthcare Provider Recommendations: Across nearly all studies, a healthcare provider’s recommendation was a key driver of vaccine acceptance. In Tunisia, 74.5% of women reported they would accept the vaccine if recommended by a provider [27]. Similarly, in Afghanistan, 86% indicated they would vaccinate if advised by a health professional and if the vaccine was freely available [36].

Geographic and Socioeconomic Disparities: Willingness to vaccinate and knowledge levels varied greatly across regions. For instance, in Afghanistan, only 23% had heard of influenza before, yet 76% expressed willingness to vaccinate, likely due to real-time awareness raised during the survey [36]. In contrast, in Pakistan, 49% had heard of influenza, but only 38% were willing to receive the vaccine [38].

Barriers to Vaccination: Commonly reported barriers included fear of side effects, misinformation, lack of time or access, absence of a recommendation by a healthcare provider, and cultural taboos. In Saudi Arabia, 32.7% of women believed the flu vaccine could cause influenza, and 16.1% believed it could cause birth defects [34]. In China, 86.3% of hesitant participants feared fetal harm from the vaccine [26].

Correlation Between Knowledge and Uptake: Several studies demonstrated that pregnant women with better knowledge of influenza and the vaccine were more likely to be vaccinated. This was evident in Ecuador, where vaccinated women had a significantly higher perception of vaccine safety and effectiveness compared to their unvaccinated counterparts [40].

These findings underscore the urgent need for targeted education, consistent healthcare provider recommendations, and systems-level strategies to improve maternal influenza vaccine uptake.

## 4. Discussion

Compared to the systematic review published in 2014 by Yuen et al. [23], which included studies up to 2013 and was limited in geographic diversity, our review incorporates 20 cross-sectional studies published since 2015 across a wider range of countries, including low- and middle-income settings such as Afghanistan, Kenya, and Kyrgyzstan. In contrast to Yuen et al., who focused primarily on determinants of vaccine uptake in high-income countries, our review offers a more globally representative picture of knowledge, attitudes, and practices regarding influenza vaccination in pregnancy [23]. Furthermore, our inclusion criteria were more stringent, focusing solely on cross-sectional studies with a defined framework and standardized methodological quality assessment using the Downs and Black checklist. These methodological refinements, combined with broader geographic scope and more recent data, provide updated insights into persistent barriers, such as misinformation, healthcare provider hesitancy, and socioeconomic disparities, and point toward targeted strategies for improving maternal vaccination rates worldwide.

The results of our review indicate that the issue of influenza awareness in the obstetric population remains a complex concern requiring subsequent thorough investigation. In addition, material conclusions and valuable contributions to the field can be drawn.

Most studies revealed that while general awareness of influenza is moderate, detailed understanding of its complications and vaccine safety is often poor. Misconceptions persist, such as confusion between antiviral and antibiotic treatments and fears of vaccine-related harm to the fetus. Our review revealed a significant disparity in the general awareness of influenza in different populations depending on the economic status of the country. The lowest awareness was noted in the Afghan population (Table 1) [36,38], whereas the highest was reported in Poland [37]. Overall, sixteen publications examined this issue [26,27,28,29,30,31,32,33,34,35,36,37,38,39,40,44]. The average general awareness ratio was 68.7% based on the most general questions about influenza knowledge included in each study. The preferred questions (in order of listing) used to estimate this value were whether participants had heard of influenza before, whether they know what the etiology of influenza is, whether they know about its high contagiousness, whether they perceive pregnant women as a risk group for contracting severe influenza, and whether they know about its possible severity. Unfortunately, only 13 of 16 studies [26,27,29,30,31,33,34,35,36,37,38,39,44] provided specific values expressed as percentages that could be utilized for calculations. Additionally, a study by Erazo evaluated pregnant women’s awareness of the severity of influenza in Ecuador [40]. However, due to the absence of a specific percentage value, the estimate can only be approximated at 80–90% based on the accompanying figure. Eleven out of these fourteen publications indicated a general knowledge of the examined population above 50% [27,29,30,31,33,34,35,37,39,40,44]. The exceptions were studies performed in Afghanistan, China, and Pakistan [26,36,38]. Afghanistan and Pakistan are the countries with the lowest gross domestic product (GDP) per capita among the 14 countries analyzed. It is noteworthy that the study in the Afghan and Pakistani populations was conducted by a similar team of researchers, with the same first author and, above all, with a similar study design. Hence, they can be compared with a lower risk of bias. The most intriguing correlation is that in the Afghan population [36], while only 23% of pregnant women had heard of influenza before, as many as 69% believe that influenza is more dangerous for pregnant women. On the other hand, in the Pakistani population [38], the general awareness of influenza was higher—49% had heard of it before—but the knowledge that women are a risk group for severe influenza was substantially lower (36%). This is also reflected in the willingness of pregnant women to vaccinate, which was substantially higher in the Afghan population (76%) than in the Pakistani population (38%). The general knowledge of the existence of influenza as a disease does not necessarily correlate with the quality of that knowledge as it is often drawn from social media or the Internet rather than from reliable sources such as healthcare providers. The high willingness of women in Afghanistan to be vaccinated may be due to the fact that they learned about the existence of influenza and the vaccine while participating in the study. This shows that the key to encouraging people to vaccinate is the involvement of healthcare professionals, who can provide high-quality knowledge both in communities with low levels of knowledge and also in those with higher declared awareness but of poor quality. Women with a greater level of knowledge about influenza demonstrated a higher likelihood of being vaccinated, as evidenced by their more frequent correct responses to specific influenza-related questions, both in countries with low and high general awareness [26,34]. A consistent finding across studies was the pivotal role of healthcare providers in influencing vaccination behavior. Pregnant women were far more likely to accept vaccination when directly advised by their obstetrician or midwife.

The persistently low awareness in these countries raises public health concerns, especially considering that maternal influenza vaccination not only protects the pregnant woman but also reduces infant morbidity. Low awareness and vaccine hesitancy in low-income settings may delay achieving global vaccination targets, increase disease burden during flu season, and complicate pandemic preparedness. Targeted educational campaigns adapted to local cultural and socioeconomic contexts are crucial to closing this gap. Socioeconomic status and geographic location significantly influence influenza vaccination rates among pregnant women. Individuals in rural areas or with lower socioeconomic status often face barriers such as limited access to healthcare facilities, lack of insurance, and reduced exposure to vaccination campaigns. For example, in the U.S., influenza vaccination rates are 4% lower among rural-residing pregnant individuals compared to their urban counterparts [45]. Addressing these disparities necessitates tailored public health strategies that consider the unique challenges of these populations.

In our opinion, even the studies claiming high general awareness of the examined population should be interpreted with caution. In a Polish study, where as many as 96.5% of women adequately identified influenza as a viral illness, only 51.1% listed antiviral drugs as a treatment for influenza, and 15.9% of women indicated antibiotics [37]. In a study conducted by Mayet in Saudi Arabia, 91.3% of women knew that influenza is highly contagious, but only 54% identified a group of pregnant women as being at higher risk of a severe course of influenza infection [34]. Moreover, despite a high general awareness, knowledge of the safety and importance of the flu vaccine during pregnancy and lactation was notably limited in this population. Only 13.1% knew it is safe during pregnancy, 16.0% during lactation, and just 19.1% were aware that flu vaccination is recommended for all pregnant women [34]. Similarly, in a Singaporean study [29], 90% of surveyed women correctly identified viruses as the cause of influenza. However, nearly half of them (46%) mistakenly selected antibiotics as the preferred treatment. Furthermore, only 46% were aware that influenza vaccination is recommended during pregnancy. To conclude, the general high knowledge of influenza among pregnant women does not necessarily reflect the quality or depth of understanding this matter, particularly regarding vaccination. Therefore, the willingness to be vaccinated remains low. Moreover, we conclude that some patients are prone to overestimating their knowledge of influenza. In a Polish study [37], most participants rated their knowledge of influenza as adequate for making a conscious decision regarding vaccination (61.9%). However, despite this confidence, only 21% reported having received the influenza vaccine during their current pregnancy and just 17.5% expressed an intention to get vaccinated.

This discrepancy between general awareness and specific, actionable understanding suggests a form of “false flu literacy”, where individuals may overestimate their knowledge and therefore undervalue medical advice. This phenomenon underscores the importance of quality-targeted communication over quantity-driven awareness campaigns. Health authorities must address not only whether women are aware of influenza but also how accurately they understand the disease and its vaccine.

The knowledge about the influenza vaccine was analyzed in 19 studies [25,26,27,28,29,30,31,32,33,34,35,36,37,38,39,40,41,42,44]. Eight studies reported a scarce level of knowledge of this topic [25,27,32,33,34,36,38,39]. Particularly low awareness regarding the existence, efficacy, and safety of the influenza vaccine during pregnancy was demonstrated among respondents from China, Saudi Arabia, Afghanistan, and Pakistan [26,34,36,38]. Chinese participants who indicated a willingness to receive the influenza vaccine during pregnancy demonstrated greater accuracy across all questions about the influenza vaccine compared to those who were hesitant. Among those who were willing to receive the vaccine, only 39.7% believed that it could transfer antibodies to the fetus and only 24.4% believed that it could reduce the spread of the influenza virus within the family. These figures were still significantly higher compared to participants who had not expressed the willingness to receive the influenza vaccine (*p* < 0.001). Among hesitant women, up to 86.3% had reservations that the administration of the influenza vaccine could be harmful to the fetus [26]. In Saudi Arabia, 63.9% of participants demonstrated poor knowledge of the flu vaccine (the median knowledge score was 5, with a range of 0–12). Awareness was notably limited regarding the safety of administering the flu vaccine during pregnancy (13.1%) and lactation (16.0%), as well as the recommendation that all pregnant women should receive the flu vaccine (19.1%). Additionally, as many as 32.7% of women believed that the influenza vaccine could be the cause of the flu and 16.1% of women believed that the flu vaccine could cause birth defects [34]. Among the Afghan women, only 11% had previously heard of the influenza vaccine. A significant 87% reported that no-one had recommended that they receive the flu vaccine during pregnancy and 81% stated that they had not received sufficient information regarding the vaccine’s efficacy and safety. Unfortunately, 60% of women were unaware of where they could access the influenza vaccine in their local area [36]. The results of the aforementioned studies indicate that significant factors contributing to these gaps in knowledge about the influenza vaccine include educational level, as well as the occupational types of participants [26,34], cultural beliefs and taboos related to pregnancy, especially among more traditional societies [26], a lack of knowledge about influenza [34], being a first pregnancy [34], and lack of information obtained from healthcare providers [36].

Safety concerns remain a predominant barrier to influenza vaccination during pregnancy. In a European systematic review, 60.4% of pregnant women believed the vaccine was unsafe during pregnancy [45,46]. These fears often stem from misinformation and a lack of clear communication from healthcare providers. Some women also harbor general anti-vaccination sentiments or believe in conspiracy theories regarding pharmaceutical companies. Combating these misconceptions requires culturally sensitive education campaigns and transparent communication about vaccine safety and efficacy.

Perceptions of vaccine effectiveness play a crucial role in vaccination decisions. Some pregnant women doubt the efficacy of the influenza vaccine, believing it may not prevent illness due to viral mutations [24,45,47]. This skepticism can deter them from getting vaccinated. Healthcare providers should emphasize the benefits of vaccination, including reduced severity of illness and protection for the newborn, to counteract these doubts.

A consistent finding across multiple studies is the profound impact of healthcare provider (HCP) recommendations on maternal influenza vaccine uptake. A meta-analysis revealed that pregnant women are 12 times more likely to receive the influenza vaccine if their HCP recommends it [45]. However, disparities exist; for instance, in Germany, only 20% of pregnant individuals reported receiving such a recommendation between 2015 and 2018. Barriers on the provider side include a lack of confidence in vaccine safety, insufficient training, and logistical challenges like vaccine storage and reimbursement issues [45]. Addressing these barriers through targeted provider education and systemic support is crucial for improving vaccination rates.

These findings implicate not only individual-level barriers but also structural gaps in healthcare systems. Where antenatal care is inconsistent, rushed, or fragmented, opportunities for maternal vaccine education are lost. Integrating influenza vaccine counseling into routine antenatal protocols and training providers to address common myths in culturally sensitive ways may significantly improve uptake.

Furthermore, an in-depth analysis of factors influencing the decision-making process of pregnant women regarding vaccination is pivotal. All the analyzed studies attempted to elucidate factors that correlated with the willingness of maternal anti-influenza vaccination [23,25,26,27,28,29,30,31,32,33,34,35,36,37,38,39,40,41,42,43,44]. Common barriers included low risk perception, fear of adverse effects, lack of time or access, and sociocultural taboos. Socioeconomic status and previous vaccination history also influenced attitudes. According to the study by Pisula et al., the factors influencing the decision to get vaccinated are average income per household and place of residence [37]. Lu et al. stated that participants’ educational level, the occupational types of participants, and the occupational types of participants’ spouses were the factors that influenced the decision to get vaccinated [26]. In a study conducted among women in Singapore, the factors identified as most significant regarding the willingness to receive the influenza vaccine were vaccination during a previous pregnancy and having private or employer health insurance that covers the cost of the vaccine [29]. Additionally, women who were personally recommended the flu vaccine during pregnancy were seven times more likely to get vaccinated than those who did not receive such a recommendation [29]. The factors identified by researchers from Saudi Arabia associated with poor uptake of the flu vaccine include poor knowledge of the flu vaccine, being below mean income, being below the mean age, unemployed status, and low education status [34]. However, multivariate analyses showed that poor uptake of the influenza vaccine is only independently associated with unemployed status after adjusting for the other dependent variables (age, income, employment status, education level, and number of pregnancies). Among American women, self-reported vaccination was associated with primigravida status, higher education, and employment in healthcare [25]. Other factors that influenced the decision regarding maternal vaccination included complications during prior pregnancies [27,35], fear of adverse effects to the fetus [26,27,28], the belief that the vaccine is not effective [28], a healthcare professional’s recommendation [27], previous anti-tetanus vaccination during childhood or adolescence [39,44], and other children in household vaccinated for influenza [44].

The insights from this review may also inform preparedness for future respiratory virus pandemics. Patterns of hesitancy and misinformation observed around influenza vaccination mirror those seen during the COVID-19 pandemic. Understanding the sociocultural and educational predictors of vaccine hesitancy in pregnancy can help governments design faster, more effective maternal immunization rollouts during future outbreaks.

This study has a few major limitations that need to be emphasized. Primarily, the discrepancy of the methodologies used by authors of each cross-sectional study in the process of data acquisition. Secondly, the questionnaires included a diverse number of differently formulated questions with various forms of assessment by the authors. Moreover, there is a heterogeneity in sample sizes as studies included between 146 and 1193 participants. Lastly, only studies published in English were included. Therefore, some important data in other languages might have been omitted. While we used the Downs and Black checklist to assess the methodological quality of included studies, we acknowledge that this tool was originally developed for evaluating non-randomized intervention studies. Although it provided a structured and reproducible means of assessing bias, confounding, and reporting quality, it does not directly address survey-specific aspects such as questionnaire development, content validity, or measurement reliability. Future reviews might consider using other tools or supplementing this with tools specifically designed for cross-sectional surveys or KAP studies to more fully assess the instrument. The strengths of our systematic review include transparent methodology, a comprehensive search across four major databases, as well as a large number of included studies conducted on diverse populations.

## 5. Conclusions

This systematic review demonstrates that pregnant women’s knowledge and perceptions about influenza and its vaccine remain suboptimal in many parts of the world. Misconceptions regarding vaccine safety, limited understanding of influenza severity, and a lack of clear communication from healthcare professionals are key contributors to low vaccination uptake. Importantly, multiple studies confirmed that recommendation by a trusted healthcare provider significantly increases vaccine acceptance [27,34,36,40]. These findings highlight the urgent need for targeted educational strategies, improved antenatal counseling, and systems-level support to ensure that maternal influenza vaccination becomes a standard and trusted component of prenatal care worldwide. According to the presented literature review, the reluctance to receive maternal vaccination often stems primarily from fears of adverse reactions or misconceptions about the vaccine’s effectiveness.

## Figures and Tables

**Table 1:** Characteristics of included studies.

Authors	Publication Year	Country	Study Population	Data Collection Method	Knowledge of Influenza	Knowledge of Vaccine	Attitude	Downs and Black Checklist Score
King et al. [25]	2020	The United States	500 pregnant women	self-administered questionairre	Not analyzed	A total of 41.2% considered the vaccine “very effective”, 49.1% “somewhat effective”, and only 37.4% considered it “very effective” for their baby.	The most common reasons against vaccination included concern about negative effects, and lack of belief in effectiveness and safety.	12 points (F.Ł.) 12 points (J.B.)
Lu et al. [26]	2024	China	1125 pregnant women	self-administered questionairre	The average correctness of answers ratio was 42.1%, with less than 32% knowing the routes of transmission and low awareness of symptoms and high-risk groups.	Only 39.7% knew that the vaccine could transfer antibodies to the fetus and 86.3% believed vaccination could harm the fetus.	A total of 93.1% of participants were reluctant to receive the influenza vaccine.	13 points (F.Ł.) 12 points (J.B.)
Dhaouadi et al. [27]	2022	Tunisia	1157 pregnant women	face-to-face interview using a standardized questionairre	A total of 86% had heard about influenza and 85.2% believed the disease is more dangerous for pregnant than non-pregnant women	A total of 35.5% considered the vaccine potentially dangerous for the fetus, and only 34% knew that it could protect the newborn	Only 36.8% of women were willing to receive maternal vaccination. Healthcare professionals (HCP) were considered the most trusted source of information on vaccines by 87.7%. Moreover, 74.5% would accept the vaccine if recommended by a HCP.	11 points (F.Ł.) 12 points (J.B.)
Khan et al. [28]	2024	Trinidad and Tobago	146 pregnant women	self-administered questionairre	The mean knowledge score was 76.6 for women during their first pregnancy and 71.01 for those who had been pregnant before.	Analyzed altogether with knowledge of influenza.	Only 26.7% of respondents had been vaccinated in a previous pregnancy. Merely 38.4% of participants had been informed about maternal vaccination by a HCP.	12 points (F.Ł.) 11 points (J.B.)
Offedu et al. [29]	2019	Singapore	500 pregnant women	self-administered questionairre	A total of 90% of surveyed women identified viruses as the cause of influenza. However, antibiotics were mostly selected as the treatment option (46%).	Only 46% were aware that influenza vaccination is recommended in pregnancy.	Self-reported influenza vaccine uptake was 9.8%. The most common reasons to get vaccinated against influenza was a recommendation by a HCP (57%).	12 points (F.Ł.) 12 points (J.B.)
Akmatova et al. [30]	2023	Kyrgyzstan	1193 pregnant women aged >18	self-administered questionairre	A total of 37.2% believed that influenza never causes hospitalization, 25.6% considered it “mild”, whereas 53.3% disagreed. A total of 83.6% considered it more dangerous in pregnancy.	A total of 37% answered that the influenza vaccine is not safe for pregnant women, and 36.6% that it is not safe for the fetus.	A total of 41.7% believed that women should receive the influenza vaccine during each pregnancy. Most common barriers for maternal vaccination were harm to the fetus, lack of effectiveness, and weakening of the immune system.	11 points (F.Ł.) 12 points (J.B.)
Ditsungnoen et al. [31]	2016	Thailand	1031 pregnant women	self-administered questionairre	A total of 70.6% of women willing to be vaccinated considered influenza more dangerous during pregnancy, and overall 62.3% thought so. A total of 76.3% believed that infection during pregnancy could harm the fetus.	A total of 82.8% and 78.2% believed that maternal influenza vaccination is beneficial to the woman and to the fetus, respectively.	Only 42% of pregnant women were willing to receive the vaccine. A total of 74% expressed the will to get vaccinated after a HCP recommendation.	12 points (F.Ł.) 11 points (J.B.)
Sağlam et al. [32]	2022	Turkey	252 pregnant women ≥ 12 weeks gestation	self-administered questionairre	The median score for questions regarding symptoms, severity, and routes of transmission was three out of five.	The median score for questions regarding benefits, immunity after vaccination, and safety was three out five.	Only 22.6% of women were willing to receive the vaccine. Reasons for declining immunization included fear of adverse reactions, lack of knowledge, and lack of time.	12 points (F.Ł.) 12 points (J.B.)
Otieno et al. [33]	2020	Kenya	507 pregnant women	face-to-face interview using a standardized questionairre	A total of 73% had heard about influenza.	A total of 60% were aware that maternal vaccination provides immunity to the infant, and 68% considered the vaccine safe.	A total of 84% of pregnant women were willing to receive the influenza vaccine.	12 points (F.Ł.) 12 points (J.B.)
Mayet et al. [34]	2016	Saudi Arabia	998 pregnant women	self-administered questionairre	A total of 91.3% were aware that influenza is highly contagious, but only 54% were aware that it is more severe in pregnant women.	Only 16% and 13.1% were aware that the influenza vacccine is safe during lactation and pregnancy, respectively	Only 18.1% took the influenza vaccine. The decision correlated with knowledge of influenza and employment status. Merely 3% had ever been offered the influenza vaccine by a HCP during pregnancy	12 points (F.Ł.) 11 points (J.B.)
Napolitano et al. [35]	2017	Italy	410 pregnant women	anonymous face-to-face interview	A total of 64.2% of pregnant women were aware that influenza poses a greater risk for pregnant women than for non-pregnant.	Only 40.9% knew that the vaccine could protect pregnant women, and merely 23.9% were aware that vaccination was recommended during pregnancy.	The majority considered maternal vaccination against influenza “not very useful” despite the fear of contracting the virus.	13 points (F.Ł.) 12 points (J.B.)
Shadid et al. [36]	2023	Afghanistan	420 pregnant women	self-administered questionairre	Just 23% of respondents had heard of influenza before, although 69% agreed that pregnant women are at higher risk.	Only 11% had previously heard about the vaccine previously, the same number of respondents had been recommended maternal vaccination	A total of 94% of the respondents trusted HCP’s recommendations, with 86% of the study population willing to get the vaccine if recommended and available free of charge.	13 points (F.Ł.) 12 points (J.B.)
Pisula et al. [37]	2022	Poland	515 pregnant women	self-administered questionairre	A total of 73% correctly identified pregnant women as the risk group for severe flu. Only 51.1% were aware that antivirals are used in the treatment of influenza, with 15.9% indicating antibiotics. A total of 96.5% knew that influenza is a virus-transmitted disease.	A total of 52% of surveyed women knew that vaccination against influenza during pregnancy is safe	Merely 21% had been vaccinated in the current pregnancy, whereas 17.5% intended to do so. A total of 46.2% were against maternal vaccination. The most common reasons for declining the vaccine included avoiding medications in pregnancy, perceived low risk of contracting influenza, or bad experiences with previous vaccinations.	13 points (F.Ł.) 13 points (J.B.)
Shadid et al. [38]	2023	Pakistan	750 pregnant women	face-to-face interviews	Only 49% had heard about influenza before. A total of 36% agreed that it is more dangerous to pregnant women.	Only 9% had heard about the vaccine. A total of 38% expressed the will to receive it, having learned about it.	A total of 58% would accept the influenza vaccine based on a WHO recommendation. The main barriers from vaccination were fear of side effects, cost of the vaccine, and allergies. A total of 65% expressed trust to HCPs.	12 points (F.Ł.) 12 points (J.B.)
Yakut et al. [39]	2020	Turkey	465 pregnant women	self-administered questionairre	A total of 52% had heard of influenza before, with only 16,8% being aware that their baby could catch influenza.	Only 3.4% of respondents were sure that influenza vaccine is safe for pregnant women.	The most significant reason for accepting maternal vaccination was a HCP recommendation.	11 points (F.Ł.) 12 points (J.B.)
Erazo et al. [40]	2021	Ecuador	842 pregnant women	self-administered questionairre	Knowledge about the influenza and the existence was significantly higher among women who reported having been vaccinated in comparison with those who reported not having been vaccinated.	More vaccinated women perceived that the influenza vaccine is safe (95.8% vs. 71.7%, respectively) and effective (68.5% vs. 61.4%, respectively) than unvaccinated women.	The most frequent reason identified as a barrier to vaccination among the respondents was the lack of recommendation/offer of the vaccine by a HCP (73.9%). Other reasons included lack of access, concern about the safety of the vaccine, and not wanting the vaccine (3.7%).	12 points (F.Ł.) 12 points (J.B.)
Maltezou et al. [41]	2019	Greece	304 pregnant women	self-administered questionairre	Not analyzed	A total of 39.5% pregnant women reported that they had already been informed about the recommendations to get vaccinated against influenza.	A total of 57% of pregnant women stated that they were willing to get vaccinated. Their obstetrician was the prevalent source of information (58%).	12 points (F.Ł.) 11 points (J.B.)
Rodríguez-Blanco [42]	2019	Spain	683 pregnant women	telephone interview	Not analyzed	A total of 91.7% had previously heard about the flu vaccine during pregnancy.	A total of 92.2% declared having received the recommendation to vaccinate. Despite that, 35.1% were not vaccinated. The most common reasons against vaccination were fear of adverse effects and lack of confidence.	11 points (F.Ł.) 12 points (J.B.)
Kang et al. [43]	2021	Korea	522 pregnant women	self-administered questionairre	Not analyzed	Not analyzed	The self-reported influenza vaccination coverage was 63.2%. A total of 43.2% did not get vaccinated as they were not aware of the importance of vaccination. Less frequent reasons against maternal vaccination included distrust of effect and fear of side effects.	13 points (F.Ł.) 12 points (J.B.)
Madewell et al. [44]	2022	Costa Rica	642 pregnant women	self-administered questionairre	Around 75% knew the routes of transmission, and over 80% knew that influenza can be a severe disease.	A total of 97% of respondents were aware of influenza vaccines, with 91.7% knowing about their safety during pregnancy.	The most common reason for accepting the vaccine was protecting one’s children. A total of 32.5% of respondents believed the vaccine could make them contract influenza or harm their infants.	12 points (F.Ł.) 12 points (J.B.)

## Data Availability

No new data were created or analyzed in this study. Data sharing is not applicable to this article.
